# Portability of short term wind power forecasting: investigating model calibration using wind power data from Ireland and UK

**DOI:** 10.1038/s41598-025-08751-3

**Published:** 2025-09-30

**Authors:** Cian Deignan, Juan Manuel González Sopeña, Bidisha Ghosh, Vikram Pakrashi

**Affiliations:** 1 UCD Centre for Mechanics, Dynamical Systems and Risk Laboratory, School of Mechanical and Material Engineering, University College Dublin, Dublin, D04 V1W8 Ireland; 2 QUANT Group, Department of Civil, Structural and Environmental Engineering, Trinity College Dublin, Dublin, D02 PN40 Ireland; 3 SOLUTE, 06006 Badajoz, Spain

**Keywords:** Wind energy, Forecasting, Portability, Variational mode decomposition (VMD), Empirical mode decomposition (EMD), Feed forward neural networks (FFNN), Energy infrastructure, Statistics

## Abstract

Wind power forecasting (WPF) models play an increasingly important role in integrating wind power into electricity systems. Portability of such models allows for quantified calibration features which can be taken from one farm and applied to another without compromising forecast accuracy. This paper investigates the portability of WPF methods by exploring the influence of model hyperparameter configurations on forecasting performance. The performance of two hybrid WPF methods are evaluated and compared, Variational Mode Decomposition & Feed Forward Neural Network (VMD-FFNN) and Ensemble Empirical Mode Decomposition & Feed Forward Neural Network (EEMD-FFNN). Supervisory Control and Data Acquisition (SCADA) data from wind farms in Ireland and the UK are utilised. The robustness and portability of the WPF methods when applied to different datasets are examined. The models demonstrated good forecasting accuracy, with the VMD-FFNN model achieving 3.42% *NMAE* error for the Irish site. For portability, the forecasting performance is found to be sensitive to two of the four model hyperparameters examined. A low number of modes used in signal decomposition, beyond a threshold of ~4 modes, is adequate for accurate prediction, although calibration is still required depending on the wind farm. Additionally, the number and variety of datasets improved model robustness.

## Introduction

### Portability of wind power forecasting

Wind power forecasting (WPF) estimates the power output of a wind turbine at a point in the future. WPF plays an increasingly vital role in delivering essential services to the energy industry, particularly with the increased share of wind generation on electricity grids. The stochastic, asynchronous and non-dispatchable nature of wind presents significant challenges in integrating additional wind generation into the energy mix. Large-scale wind penetration means that grid operation, meeting reserve and ancillary requirements, meeting electricity demand, and maintaining grid stability is more complex^1^. These challenges manifest across a variety of timescales, from a few seconds to weeks or months ahead of time^2^. Improved WPF contributes to the improvement of the flexibility of the electricity system and allows grid operation with a greater share of renewables.

While WPF accuracy remains an important question, developing a reliable model for each turbine or wind farm and calibrating key parameters of such models can often be unreasonably laborious and computationally intensive. However, it is also observed that energy generation is typically linked to the wind resources along with the types of turbines and their placement in a farm. Consequently, similar farms should have some similarities in their models, which can possibly be captured through the similarities of some of the calibrated parameters. The possibility of being able to use some of the parameters of one farm on another is of particular interest under such circumstances, since such an approach provides rapid estimates of production with minimal change or measurement. It also allows for classification of similar sites and take a centralized decision based on classes of models that fit a certain type of farm, rather than individualized and disconnected estimates for each farm. This possibility of using quantified features from one location to another, without losing WPF accuracy, is considered here as *portability*.

In this paper, the term * portability * refers to the suitability of the use of model hyperparameters calibrated for one farm to another farm without a reduction in WPF performance. With machine learning dominating several data analytics approaches, the approach considered in this paper also allows for choice of robust architecture of WPF models with lower sensitivity against changes in model hyperparameters. In this context, this paper also investigates which hyperparameters have a significant influence on WPF performance and which hyperparameter calibrations optimise forecasting accuracy.

Although the idea of portability is understood and discussed in the context of the wind engineering community, WPF methods and their tests around portability are relatively unexplored, despite some recent papers examining hyperparameter optimization of WPF models^3,4^. Recent studies have also examined the robustness of WPF methods, an area related to portability^5,6^. A gap still exists in addressing portability, for instance, by examining the robustness of models in generalizing to new datasets. The reason for such paucity in investigating portability lies in the lack of comparative studies on real datasets at a farm level. This paper addresses this gap by investigating two efficient WPF methods for two different farms in Ireland and the UK with a range of variability in their fundamental behaviour.

The results first present a benchmark example and a framework to establish the portability of models and provide guidance in terms of performance levels and choice of hyperparameters to enhance reliable farm-level wind power predictions. This approach is expected to lead to a wider uptake of such studies, eventually leading to hyperparameter calibrations on smaller micro-zones and eventually to medium or larger regions. This in turn can lead to reliable, consistent and rapid estimates of WPF. An approach with the idea of portability also establishes similarities of underlying signatures of wind power at a farm level as an outcome of complex geophysical interactions, wind resources and the turbines.

### Applications and contribution

Wind power forecasts can be used as input for economic load dispatch and load increment planning, allowing for transmission system operators (TSO) to optimize the allocation of generating units to match demand at minimum cost while satisfying system reliability constraints^7,8^. Accurate WPF can also help mitigate the need for wind curtailment^9^. Additionally, WPF decision tools can be used to identify possible day-ahead network congestion and voltage violations along with assistance in specifying operational reserve requirements for the coming hours or days^10^. These applications of WPF are particularly useful for TSOs aiming to increase allowable levels of instantaneous renewable generation on electricity grid systems^11^.

WPF is also a key to creating competitive and efficient electricity markets; asset owners, suppliers, and energy traders can leverage them to support financial decision-making. The accuracy of WPF is significantly linked to such decisions^1^. Changes to WPF in time horizons of 3–6 hours can provide information influencing decision making in Day-Ahead and Intra-Day markets. For example, an examination of WPF-informed trading on the Danish Intra-Day electricity market demonstrated an improved financial trading performance^12^. Additionally, market participation regulation costs associated with balancing actions can be reduced by integrating probabilistic forecasts into market participation strategies^13^. Consumers also benefit as competitive pricing at electricity markets tends to lead to the delivery of good value electricity to consumers.

Successful outcomes in many of the applications discussed are underpinned by highly accurate WPF for significant numbers of wind farms spread over extensive geographical regions. The contribution of this paper lies in the potential to unlock the scaling of forecasting accuracy from single wind farms to groups of farms or larger regions. The portability of WPF models can achieve this by applying model hyperparameter calibrations in the correct context to optimise forecasting performance. This contribution has the potential to be magnified with additional comparable studies on other farms; with a wider variety of datasets, wind turbine models and site-specific conditions, useful hyperparameter calibrations on wind farm groups or larger regions will become apparent. The applications in grid operation and electricity markets discussed earlier can all benefit as a consequence of improved WPF performance.

## Data

### Data overview

Supervisory Control and Data Acquisition (SCADA) datasets from three wind farms are used, including an Irish wind farm and two wind farms in the UK. The Irish raw dataset used in this project is confidential, while two datasets from the UK (Penmanshiel and Kelmarsh farms) are available under a CC-BY-4.0 open source license^14^ and are available in Zenodo, developed under the European OpenAIRE program^15^. Before analysis, the quality of data is improved first by investigating aspects like missing data entries, incorrect data formatting and misaligned labelling.

### Data preprocessing: data cleaning & missing value imputation

This step ensures that the number of datapoints in a time series corresponds to the time period of the dataset. For instance, a one-year dataset with 1-hour resolution should have 8,760 datapoints. Additionally, date and time index labels are checked to ensure that they are contiguous. Missing values can often be found in raw SCADA data as a result of occasional errors from measurement instruments or weather effects such as icing. Missing values are an important consideration as they can skew performance metrics. The missing values in the Penmanshiel and Kelmarsh farms were imputed with estimated values before forecasting. A k-Nearest Neighbours (k-NN) strategy is used for data imputation^16^, which uses a weighted average of adjacent actual values to create missing value estimates^17^. The k-NN imputation approach can be memory-intensive; processing the data in batches can be effective in mitigating against this. There is further scope to improve the quality of imputed data using other new encoding methods^18^ but the k-NN strategy is a well established, robust and reliable approach around which the idea of model portability can be investigated.

Occasionally, turbines or entire wind farms may experience a significant fault or maintenance issue, leading to an extended period of zero power output. These prolonged flat lines in a power dataset should be excluded from WPF modeling. The datasets do not have such extended periods of no power generation.

### Irish wind farm dataset

**Fig. 1:**
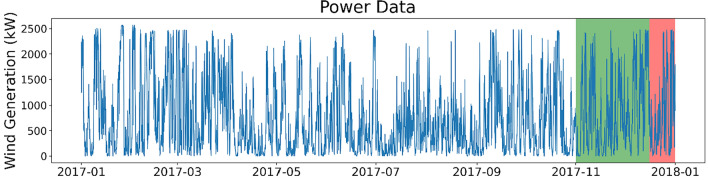
Power dataset for the Irish wind farm (1-hour resolution). The training, validation, and test datasets are shown in white, green, and red, respectively.

The dataset taken from Ireland consists of the total output power of a wind farm measured every 10 minutes between January 2017 and June 2019 inclusive. Five 12-month long datasets are used for analyses. One of these datasets, for the year of 2017, is described in Fig. 1. Three of these datasets are used for model training.

The datasets are split into training, validation, and testing sets for model training and subsequently evaluating the forecasting accuracies (shown in colour divisions in Fig. 1). The ratio of training, validation, and testing split is chosen to mirror the divisions used in a pre-existing benchmark (see Validation of Methodology). The Irish dataset has ~10 months for training, 45 days for validation, and 16 days for testing. The datasets are resampled at a lower resolution (1-hour) as well as at the higher resolution (10-min) at which it is measured. This is done to examine the influence of data resolution on forecasting accuracy. This dataset has already been cleaned so no data cleaning or missing value imputation is required at this stage.

### Penmanshiel wind farm dataset

The second dataset is taken from the Penmanshiel wind farm in Scotland. Penmanshiel wind farm is located to the east of Edinburgh close to the Scottish Borders. SCADA data from the wind farm is available online via a CC-BY-4.0 open source license^19^. The wind farm consists of fourteen Senvion MM82/2050 turbines, each with a rotor diameter of 82m. The total nominal power of the wind farm is 28.7 MW. The dataset contains the aggregated total output of the wind farm between January 2017 and December 2019, inclusive, with a 10-minute resolution. This dataset is also resampled at an hourly resolution.

**Fig. 2:**
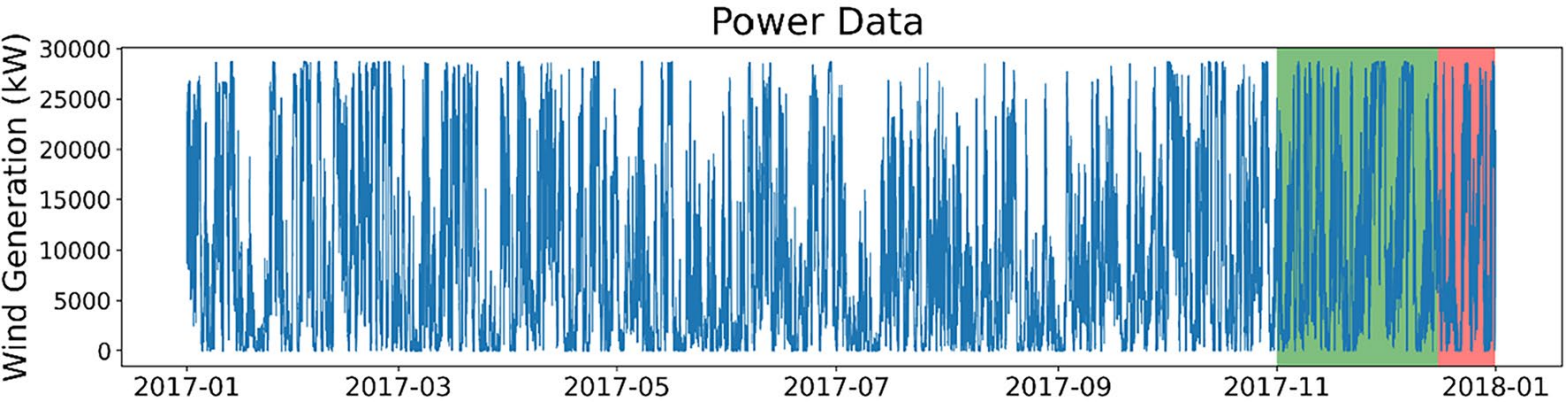
Power dataset for Penmanshiel wind farm (1-hour resolution). The training, validation, and test datasets are shown in white, green, and red, respectively.

Five 12-month long datasets are sampled from the main dataset, and a plot for the year of 2017 is shown in Fig. 2. Three of these datasets are used for model training. Note that there are a small number of negative power output values present in this data (Table 1), which can be attributed to a small amount of stationary consumption during low wind, reactive power management, or sudden drops in wind speed.

The size of the dataset used from Penmanshiel wind farm is the same (i.e. same number of values) as the Irish dataset and the split between training, validation and testing is also the same (Table 1). This allows for better comparison between the two sites since data quantity, particularly the weighting of training, validation and test splitting, has a strong influence on the accuracy of forecasting outcomes.

Of the ~2.2 million data points in the dataset (prior to turbine output aggregation for the whole wind farm) 12,981 missing values were absent, accounting for ~0.6% of the total dataset. These missing values were imputed with estimated values using a k-Nearest Neighbours (k-NN) strategy. After missing value imputation, the turbine outputs were aggregated to a total wind farm output at each time stamp, prior to being used for forecasting.

### Kelmarsh wind farm dataset

**Fig. 3:**
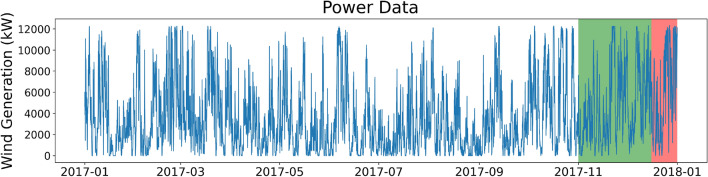
Power dataset for Kelmarsh wind farm (1-hour resolution). The training, validation, and test datasets are shown in white, green, and red, respectively.

The third dataset is taken from Kelmarsh wind farm, situated in Northamptonshire, East Midlands, England. SCADA data from the wind farm is available online via a CC-BY-4.0 open source license^20^. The wind farm consists of six Senvion MM92 turbines, each with a rotor diameter of 92.5m. The total nominal power of the wind farm is 12.3 MW. The dataset contains the aggregated total output of the wind farm between January 2017 and December 2019, inclusive, with a 10-minute resolution; this is the same timeframe as the Penmanshiel dataset. The dataset is also resampled at an hourly resolution. Five 12-month long datasets are sampled from the main dataset, one of these datasets for the year of 2017 is shown in Fig. 3. Three of these datasets are used for model training.

The Kelmarsh wind farm dataset is also the same size as the Penmanshiel and the Irish wind farm dataset along with the same split between training, validation and testing (Tables 1).

15,005 of the ~946,000 data points in the dataset (prior to turbine output aggregation for the whole wind farm), were missing initially, accounting for ~1.6% of the total dataset. This was ~1% more than the Penmanshiel dataset. These values were imputed via k-Nearest Neighbours (k-NN) and subsequently aggregated to a total wind farm output at each time stamp. Table 1 summarises the key statistics of the datasets used in this paper.

**Table 1:** Data summary statistics.

Resolution	Irish dataset		Penmanshiel dataset		Kelmarsh dataset	
	1-hour	10-min	1-hour	10-min	1-hour	10-min
Total data points	8760	52560	8760	52560	8760	52560
Training	7296	43776	7296	43776	7296	43776
Validation	1080	6480	1080	6480	1080	6480
Test	384	2304	384	2304	384	2304
Mean (kW)	763.17	763.18	9715.21	9715.20	3738.69	3738.70
Std (kW)	707.73	717.61	8884.52	9053.55	3309.41	3380.13
Q1 (kW)	177.0	170.3	1983.0	1851.2	1079.0	1026.1
Q2 (kW)	526.0	517.8	6939.5	6826.3	2791.5	2742.3
Q3 (kW)	1199.3	1203.9	15856.0	16156.1	5636.8	5632.7
Max (kW)	2571.0	2572.4	28696.0	28703.5	12333.0	12357.0
Min (kW)	0	0	-79.0	-79.0	-38.0	-38.0

## Methodology

Forecasts were obtained using the VMD-FFNN and EEMD-FFNN methods. Prior to this, the implementation of these methods were validated against existing benchmarks (see Validation of Methodology) where these methods were observed to be efficient and resilient. Subsequently, an assessment of the portability of the forecasting methods is carried out (see Portability of Results).

### WPF theory

A significant amount of existing research has explored various WPF methods^21,22^. The two methods used in this paper, VMD-FFNN and EEMD-FFNN, were selected as they are well documented and accurate. Additionally, the performance metrics used are shared publicly, which allows reproducibility of the presented results.

As WPF technologies have developed, hybrid methods using a combination of different models are observed to achieve optimal forecasting results^21,23,24^. Decomposition-based hybrid models decompose wind power time series into a superposition of simpler signals (modes) before being input into an Artificial Neural Network (ANN). Signal decomposition is carried out during pre-processing to obtain insights into underlying system, while reducing the effects of non-stationarity, removing noise and other signal artefacts^25^. Several decomposition-based techniques have been applied to WPF in the literature, including wavelet transform^26^, Variational Mode Decomposition (VMD)^27^, and Ensemble Empirical Mode Decomposition (EEMD)^28^.

EEMD^29^ is an adaptation of the Empirical Mode Decomposition (EMD) method^30^. It is a recursive algorithm that decomposes a time series into a set of modes. The benefit of decomposing the power signal into modes is that linearity can be utilised to improve the processing of these signals, enhancing forecasting accuracy. EEMD uses multiple trials of the EMD algorithm where the original data is mixed with Gaussian white noise. The algorithm then shifts towards the most persistent and meaningful part of the signal.

VMD is a non-recursive signal processing method where the modes are estimated concurrently; it is useful for decomposing complex non-stationary signals^31^. The decomposition process involves applying the Hilbert transform to the signal, shifting each mode’s frequency spectrum to base bands using estimated centre frequencies, and identifying the bandwidth of each mode using the H1 Gaussian smoothness of the signal^32^. The variational problem is then solved using the alternating direction method of multipliers (ADMM) algorithm^33,34^. In this study, decomposed signals obtained using VMD and EEMD are fed into an ANN, specifically a Feed Forward Neural Network (FFNN). The output signal is subsequently recompiled into the form of the original signal (see Experimental design: forecasts & errors).

The VMD-FFNN and EEMD-FFNN methods are used to obtain point forecasts of the power output (i.e. single-value estimates at a given timestep in a time series). Probabilistic forecasts are also obtained in this study, as they provide significant information about forecasting accuracy. Probabilistic forecasts describe the uncertainty associated with a given wind power forecast; this is usually defined by a prediction interval. The quantile regression method estimates the uncertainty of a forecast through a set of quantile estimates. This method has been used to create prediction intervals using wind farm data from Vikna, Norway^35^. The quantile regression method is used to construct prediction intervals in this study.

### Experimental design: forecasts & errors

**Fig. 4:**
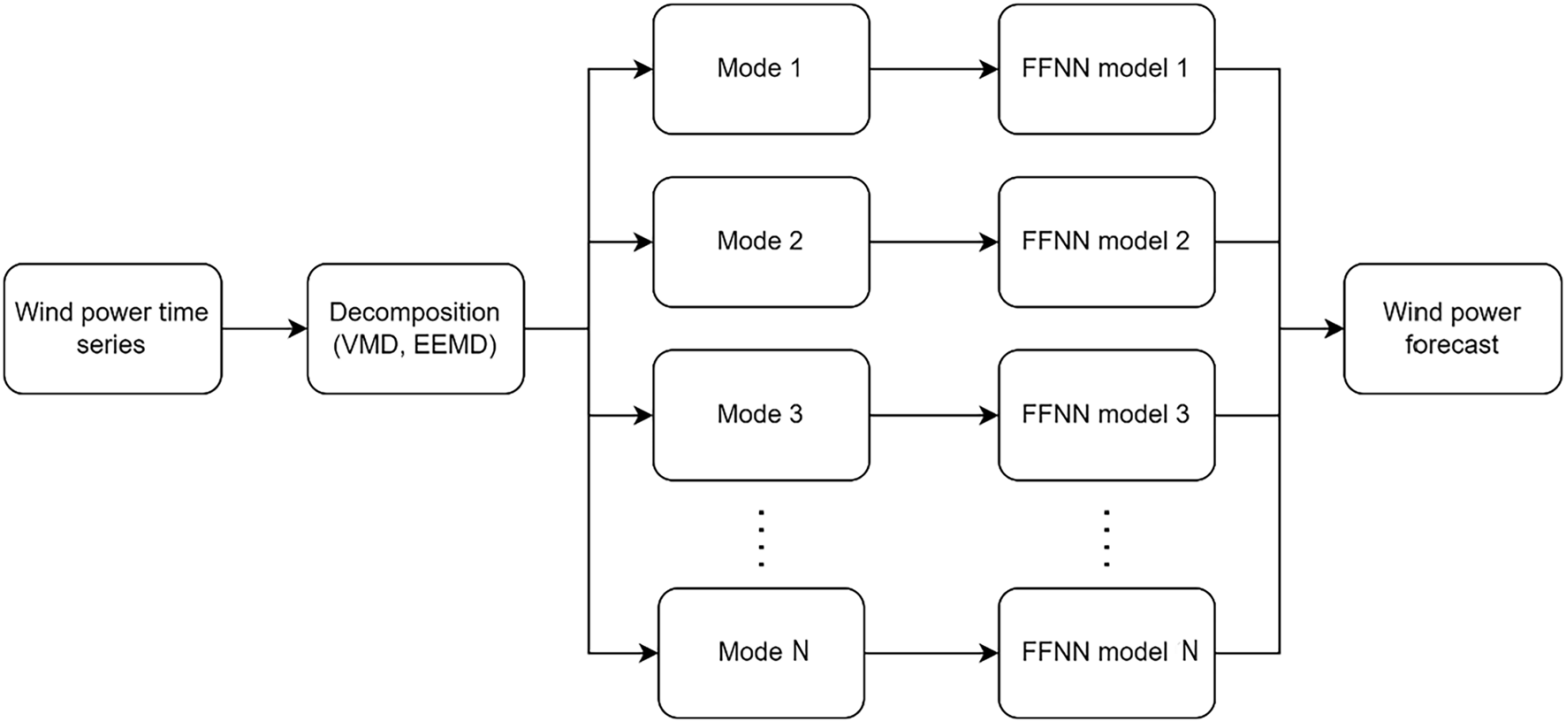
Flowchart of hybrid decomposition-based WPF method.

The two WPF methods used can be categorised as hybrid decomposition-based forecasting methods. These methods first decompose the normalised wind power time series into six simpler signals, called modes, via VMD and EEMD. The modes are fed into a feed forward neural network FFNN and a forecasting model is built for each mode. The final wind power forecast is produced by aggregating the forecasts for each mode (Fig. 4).

The methods are used for both high- (10 min) and low-resolution (1 hour) datasets, where the low resolution dataset is obtained through sub-sampling of the higher resolution datasets. The final prediction consists of a single power output estimate at each timestep, along with upper and lower bounds of the prediction interval, respectively. The boundaries of the prediction intervals are estimated at a 95% confidence level using a quantile regression method that uses an asymmetric loss function (or pinball function)^36,37^ (see WPF Theory).

The low-resolution data *only* is used for probabilistic forecasting, noting that a previous study indicated that the hybrid methods like VMD or EMD may not provided higher performance with a higher resolution of data since the prediction intervals can be too wide to be informative^38^. The issue may be related to the high frequency components in the high resolution data, the number of steps ahead in the prediction horizon, or the fact that the only input to the forecasting model is power data, which is typically the variable available. Prediction intervals produced with low-resolution data are shown to be reasonably narrow and informative, therefore the focus is on a 1-hour resolution.

Point estimate and prediction interval metrics are used to evaluate errors associated with the forecasting methods. Six point estimate metrics and five probabilistic metrics are considered (Table 2). These metrics are described in detail in the Error Metrics section of this paper.

The forecasts were made at a six-hour ahead forecast horizon. A multi-step ahead forecasting technique was used for this purpose, which estimated the next *H* steps $[y_{t+1},\ldots,y_{t+H}]$ in a time series^31^. In this work, the high-resolution data is forecasted 36 timesteps ahead, and the low-resolution data is forecasted 6 timesteps ahead. This forecast horizon was chosen as it is a useful horizon for energy trading in electricity markets and for electricity grid operation. The multi-step ahead forecast is computed using the Multiple-input Multiple-output method (MIMO), which produces a vector with the full sequence of future outputs of the time series (Eq. 1).

- *Multi-step ahead forecasting*
  1 $$\begin{aligned} {[}y_{t+1},\ldots,y_{t+H}] = f(y_{t},\ldots,y_{t-d+1}) \end{aligned}$$

**Fig. 5:**
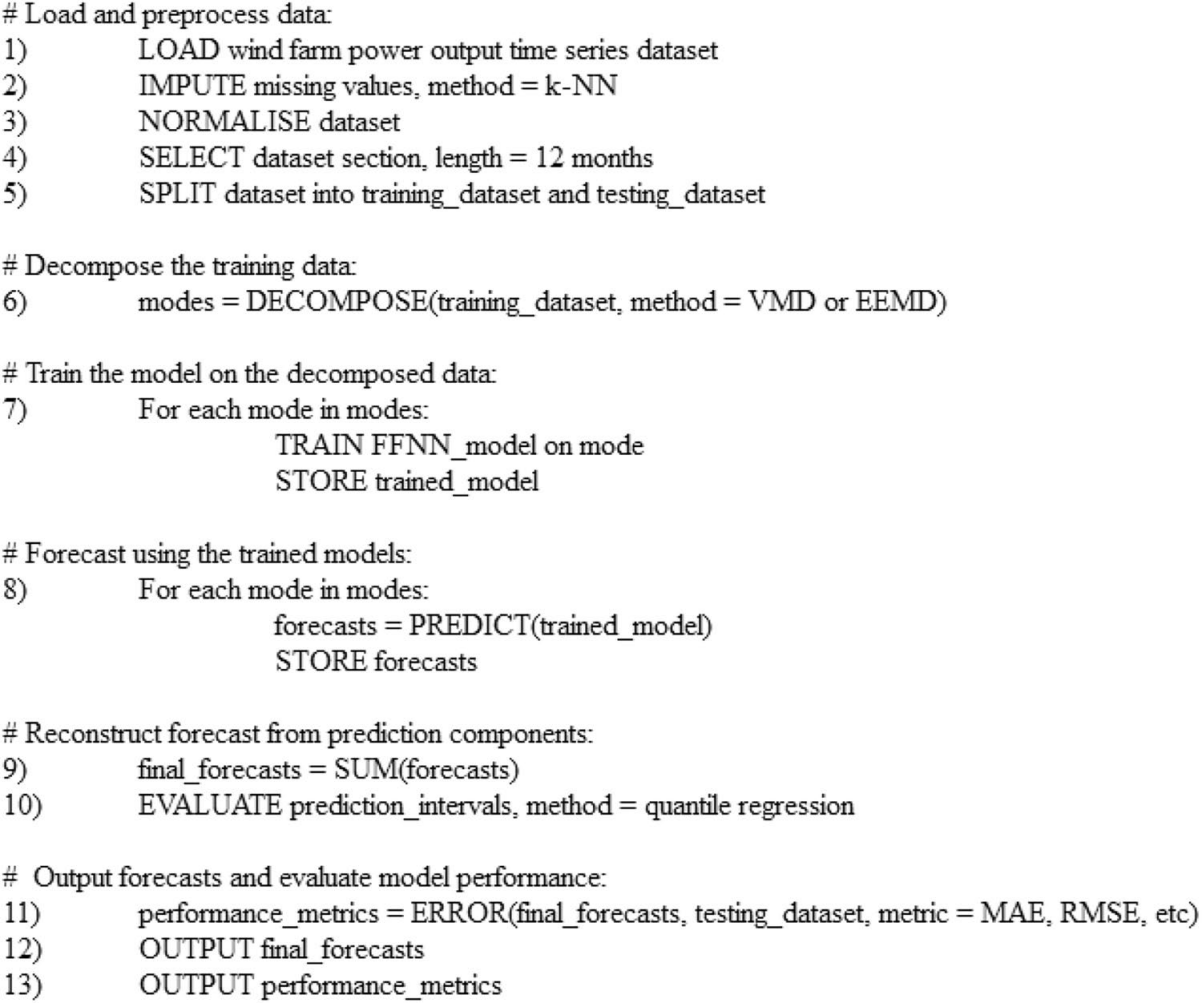
Pseudocode of the WPF method.

The VMD algorithm is implemented using the *vmdpy* library^39^, EEMD with the *PyEMD* library^40^, and *Keras* with *Tensorflow* backend is used for the FFNN^41,42^. Pseudocode for the WPF method is shown in Fig. 5. This is a simplified version of the steps used in the forecasting method to aid the readers understanding of the process.

### Error metrics

It is vital to understand how the accuracy of a WPF method is evaluated to assess model accuracy. There are several metrics associated with both deterministic and probabilistic forecasts (Table 2). Deterministic metrics describe the accuracy of point forecasts, which are single values of power output at a given timestep. Probabilistic forecasts describe the uncertainty associated with a given point forecast, or the probability that the point estimate falls within a given range. The uncertainty is usually described by a prediction interval, which is defined by an upper and a lower bound, respectively. Many of the performance metrics are normalised, which means that the differences in wind farm capacity do not affect the comparison of different datasets and the metrics are comparable.

**Table 2:** Performance Metrics.

Deterministic		Probabilistic	
*NMAE*	Normalised mean absolute error (%)	*PICP*	Prediction interval coverage probability (%)
*NRMSE*	Normalised root mean square error (%)	*PINAW*	Prediction interval normalised average width (%)
*MAPE*	Mean absolute percentage error (%)	*CWC*	Coverage width-based criterion
*NBias*	Normalised Bias (%)	*ACE*	Average cover error
*NSDE*	Normalised standard deviation error (%)	*IS*	Interval sharpness
*IA*	Index of agreement		

#### Error metrics of deterministic forecasts

The accuracy of deterministic forecasts are evaluated using several metrics. The most commonly used metrics are as follows^31^:

- Mean absolute error (*MAE*):
  2 $$\begin{aligned} \textit{MAE} = \frac{1}{N} \sum _{i=1}^{N} |\hat{y}_i - y_i| \end{aligned}$$
- Root mean square error (*RMSE*):
  3 $$\begin{aligned} \textit{RMSE} = \sqrt{\frac{1}{N} \sum _{i=1}^{N} (\hat{y}_i - y_i)^2} \end{aligned}$$
- Mean absolute percentage error (*MAPE*):
  4 $$\begin{aligned} \textit{MAPE} = \frac{1}{N} \sum _{i=1}^{N} \left| \frac{y_i - \hat{y}_i}{y_i} \right| \cdot 100\% \end{aligned}$$
- Standard deviation error (*SDE*):
  5 $$\begin{aligned} \textit{SDE} = \sqrt{\frac{1}{N} \sum _{i=1}^{N} (\epsilon _i - \bar{\epsilon })^2} \end{aligned}$$
- Bias:
  6 $$\begin{aligned} \textit{BIAS} = \frac{1}{N} \sum _{i=1}^{N} y_i - \hat{y}_i \end{aligned}$$
- Index of agreement (*IS*):
  7 $$\begin{aligned} \textit{IA} = 1 - \frac{\displaystyle \sum _{i=1}^{N} (y_i - \hat{y}_i)^2}{\displaystyle \sum _{i=1}^{N} (\left| \hat{y}_i - \bar{y} \right| + \left| y_i - \bar{y} \right| )^2} \end{aligned}$$

Where $y_{i}$ is the actual value, $\hat{y_{i}}$ is the predicted value, *N* is the number of samples, $\bar{y}$ is the mean value of the real values, $\epsilon _{i} = y_{i} - \hat{y_{i}}$ is the prediction error (or residual), and $\bar{\epsilon }$ is the average value of the errors^31^.

The *MAE* is the average value of the prediction errors in absolute values (Eq. 2). The *RMSE* describes the standard deviation of the prediction errors (Eq. 3). Normalised versions of both metrics (*NMAE* and *NRMSE*) are also commonly used and are calculated by dividing their non-normalised equivalent by the mean of the actual values. The *MAPE* describes the accuracy as a percentage of the error (Eq. 4) and is essentially a percentage equivalent of the *MAE*.

Prediction errors have inherently random components and systematic errors. The *SDE* (Eq. 5) identifies the random component, and the *Bias* (Eq. 6) identifies the systematic component. Finally, the index of agreement (Eq. 7) describes the degree to which predictions are without error, taking a value of one when predictions match the actual values exactly, and zero when there is no agreement between the predictions and actual values^43^.

#### Error metrics of probabilistic forecasts

The accuracy of probabilistic forecasts are evaluated using several different metrics^31^. Where the prediction interval is defined at a certain level of confidence $\alpha$, the probability that the actual wind power output $y_{i}$ lies within the prediction interval is described as the prediction interval nominal confidence (*PINC*) (Eq. 8):

- Prediction interval nominal confidence (*PINC*):
  8 $$\begin{aligned} \textit{PINC} = 100(1 - \alpha )\% \end{aligned}$$

Reliability and sharpness are two additional properties of prediction intervals. Reliability is a measure of how often point forecasts fall within the prediction interval. Prediction interval coverage probability (*PICP*) is a metric for reliability (Eq. 9). Sharpness describes the width of the prediction interval; the sharpness of a prediction interval is often described using the prediction interval normalised average width (*PINAW*) metric (Eq. 10).

- Prediction interval coverage probability (*PICP*)
  9 $$\begin{aligned} \textit{PICP} = \frac{1}{N} \sum _{i=1}^{N} c_i \quad \textit{where} \quad c_i = {\left\{ \begin{array}{ll} 1, & \text {if } y_i \in \hat{I}_i^\alpha \\ 0, & \textit{otherwise} \end{array}\right. } \end{aligned}$$
- Prediction interval normalized average width (*PINAW*)
  10 $$\begin{aligned} \textit{PINAW} = \frac{1}{NR} \sum _{i=1}^{N} \hat{I}_i^\alpha \quad \textit{where R is target variable range} \end{aligned}$$

Ideally, the width of the prediction interval is as narrow as possible while maintaining a high level of reliability. The coverage width-based criterion (*CWC*) (Eq. 11) is a useful metric for evaluating the interaction of reliability and sharpness as it combines both the *PICP* and *PINAW*.

- Coverage width based criterion (*CWC*)
  11 $$\begin{aligned} \textit{CWC} = \textit{PINAW}\left[ 1 + \gamma (\textit{PICP})e^{-\eta (\textit{PICP} - \mu )}\right] \end{aligned}$$
  *where* $$\gamma (\textit{PICP}) = {\left\{ \begin{array}{ll} 0, & \text {if } \textit{PICP} \ge \mu \\ 1, & \text {if } \textit{PICP} < \mu \end{array}\right. }$$

The average coverage error (*ACE*) is another metric for describing the reliability of a prediction interval (Eq. 12). Smaller *ACE* numbers indicate a smaller difference between the *PICP* and *PINC*, indicating a more reliable prediction interval.

- Average cover error (*ACE*)
  12 $$\begin{aligned} \textit{ACE} = \textit{PICP} - \textit{PINC} \end{aligned}$$

The interval sharpness (*IS*) is a measure of the sharpness of the prediction interval (Eq. 13). This metric identifies narrower intervals as estimates that fall outside the interval are penalised.

- Interval sharpness (*IS*)
  13 $$\begin{aligned} IS = \frac{1}{N} \sum _{i=1}^{N} b_i \end{aligned}$$
  *where*
  $$b_i = {\left\{ \begin{array}{ll} -2\alpha \hat{I}_i^\alpha - 4\left( \hat{L}_i^\alpha - y_i\right) , & \text {if } y_i < \hat{L}_i^\alpha \\ -2\alpha \hat{I}_i^\alpha , & \text {if } y_i \in \hat{I}_i^\alpha \\ -2\alpha \hat{I}_i^\alpha - 4\left( y_i - \hat{U}_i^\alpha \right) , & \text {if } y_i > \hat{U}_i^\alpha \end{array}\right. }$$

### Validation of methodology

Prior to obtaining forecasts using the open-source datasets, the implementation of the forecasting method was validated by a VMD-FFNN model and an EEMD-FFNN model and comparing against an existing benchmark^31^. It was observed that the VMD-FFNN method was less accurate than the original results for the low resolution forecasts, but more accurate for the high resolution forecasts. Additionally, the *NMAE* and *NRMSE* are lower (higher performing) for the EEMD-FFNN method for both resolutions, and the prediction intervals for both methods were not as narrow as in the original results in the existing benchmark. While the reproduced results cannot align precisely with the original benchmarks, the results match closely, validating the forecasting methodology and their use for subsequent analysis in this paper.

#### Deterministic performance metrics

**Fig. 6:**
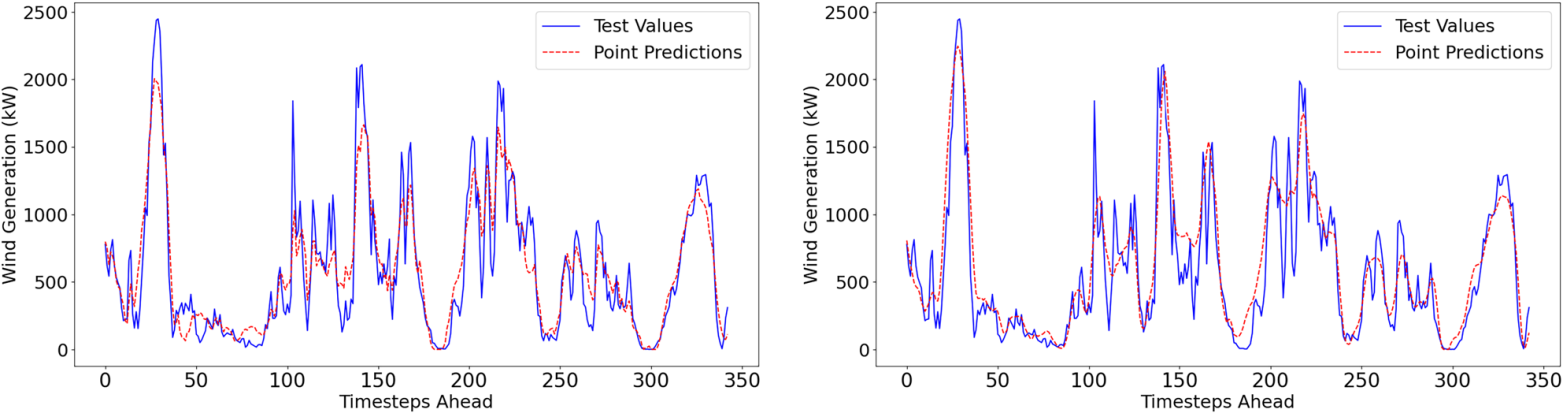
Point estimates with actual test values for VMD-FFNN (left) and EEMD-FFNN (right) with 1-hr resolution.

The reproduced deterministic results are displayed in Table 3. Visualisations of the point predictions and the actual values for both forecasting methods are displayed in Fig. 6.

**Table 3:** Reproduced Point Estimate Results.

Forecast horizon = 6h ahead						
Method	NMAE(%)	NRMSE(%)	MAPE(%)	NBias(%)	NSDE(%)	IA
Low resolution dataset (1-hr resolution)						
VMD–FFNN	5.496	7.475	26.49	-0.12	7.47	0.9592
EEMD–FFNN	5.977	8.350	29.19	-1.94	8.12	0.9542
High resolution dataset (10-min resolution)						
Method	NMAE(%)	NRMSE(%)	MAPE(%)	NBias(%)	NSDE(%)	IA
VMD–FFNN	5.489	8.057	25.56	0.42	8.05	0.9546
EEMD–FFNN	7.975	10.381	30.27	-2.43	10.09	0.9194

#### Probabilistic performance metrics

**Fig. 7:**
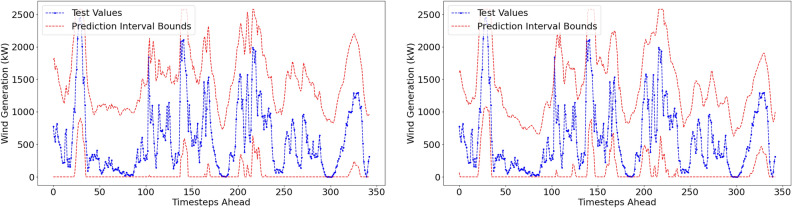
Prediction Intervals with actual test values for VMD-FFNN (left) and EEMD-FFNN (right) with 1-hr resolution.

The reproduced probabilistic results are displayed in Table 4. Visualisations of the prediction intervals and the actual values for both forecasting methods are displayed in Fig. 7. Prediction intervals are made at a 6-hour ahead time horizon at a confidence level of 95%.

**Table 4:** Reproduced Prediction Interval Results.

Confidence level: 95%					
Forecast horizon = 6h ahead					
Method	PICP (%)	PINAW (%)	CWC	ACE	IS
Low resolution dataset (1-hr resolution)					
VMD–FFNN	100.00	57.33	0.573	0.050	-147.92
EEMD–FFNN	99.71	52.61	0.526	0.047	-137.22
High resolution dataset (10-min resolution)					
Method	PICP (%)	PINAW (%)	CWC	ACE	IS
VMD–FFNN	100.00	85.99	0.860	0.050	-221.87
EEMD–FFNN	100.00	86.21	0.862	0.050	-222.43

## Results

Following method validation, the forecasting methods are then applied to two new wind power datasets from Penmanshiel and Kelmarsh wind farm. The accuracy of the forecasting methods were assessed using both deterministic and probabilistic performance metrics. The forecasting experimental design is described in detail in 3.3 Experimental design: forecasts & errors.

### Deterministic performance metrics

#### Penmanshiel wind farm: deterministic performance

**Fig. 8:**
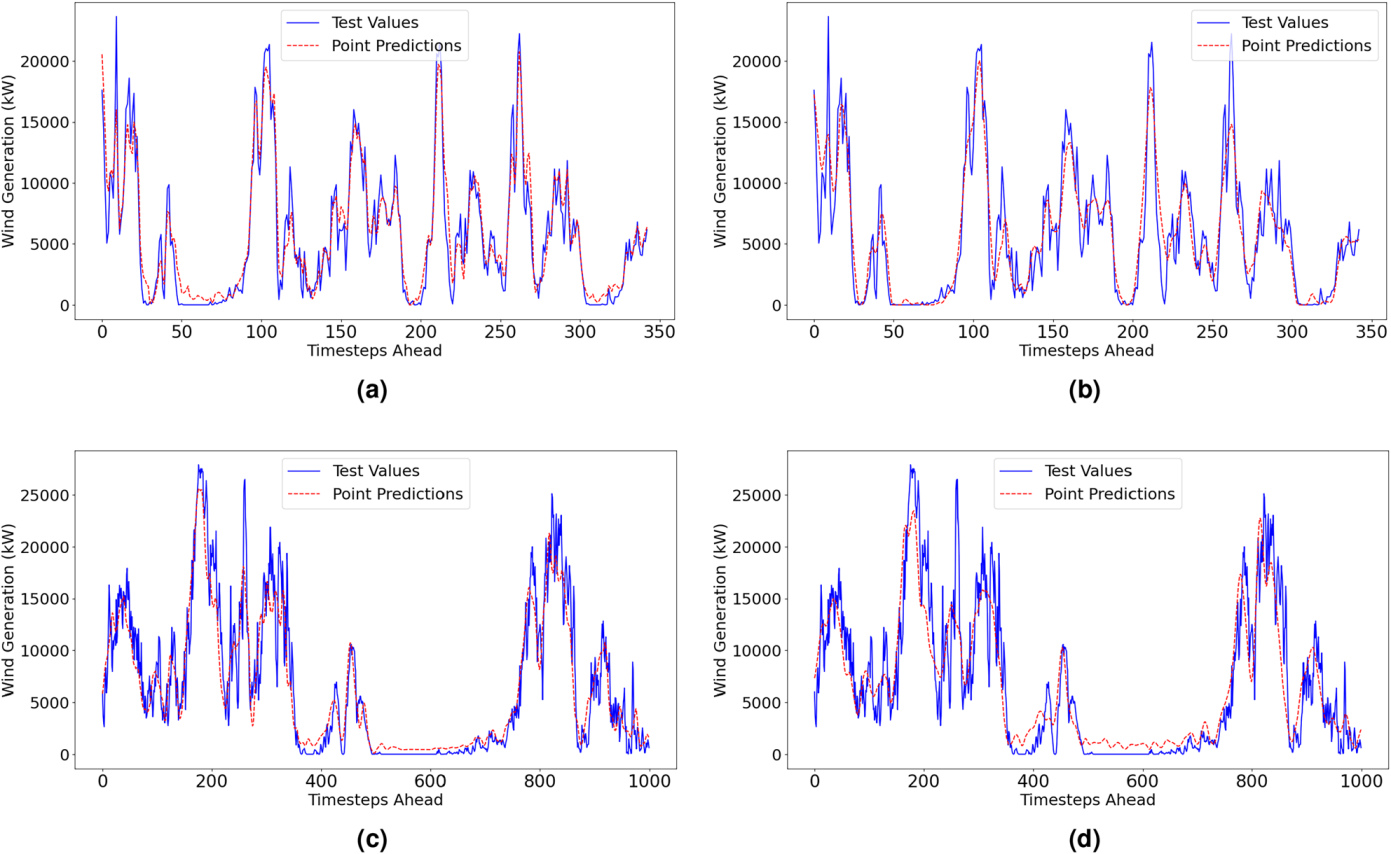
Deterministic results for the Penmanshiel dataset.

Deterministic forecasting metrics for the Penmanshiel wind farm dataset are shown in Table 5. Visualisations of the point estimate performance are shown in Fig. 8. The forecasts indicate low values for *NMAE*, *NRMSE*, *MAPE* and the values close to 1 for *IA*. Overall, the performance metrics for both methods are similar to those from the Irish dataset used for method validation. This is indicative of a robust modelling methodology that can maintain reliable results while adapting to different datasets.

The most notable difference in performance is that the VMD-FFNN method with low-resolution data was notably more accurate for the Penmanshiel dataset than the original Irish dataset. The *IA* rose by 0.02, the *NMAE* dropped by ~1.5%, and the *MAPE* reduced by ~6%.

**Table 5:** Point Estimate Results—Penmanshiel Wind Farm.

Forecast horizon = 6h ahead						
Method	NMAE(%)	NRMSE(%)	MAPE(%)	NBias(%)	NSDE(%)	IA
Low resolution dataset (1-hr resolution)						
VMD–FFNN	3.881	5.322	20.29	-0.91	5.24	0.9780
EEMD–FFNN	5.097	7.353	29.00	-0.08	7.35	0.9557
High resolution dataset (10-min resolution)						
Method	NMAE(%)	NRMSE(%)	MAPE(%)	NBias(%)	NSDE(%)	IA
VMD–FFNN	5.691	8.022	26.07	0.47	8.01	0.9558
EEMD–FFNN	6.819	9.251	28.61	-0.07	9.25	0.9387

#### Kelmarsh wind farm: deterministic performance

**Fig. 9:**
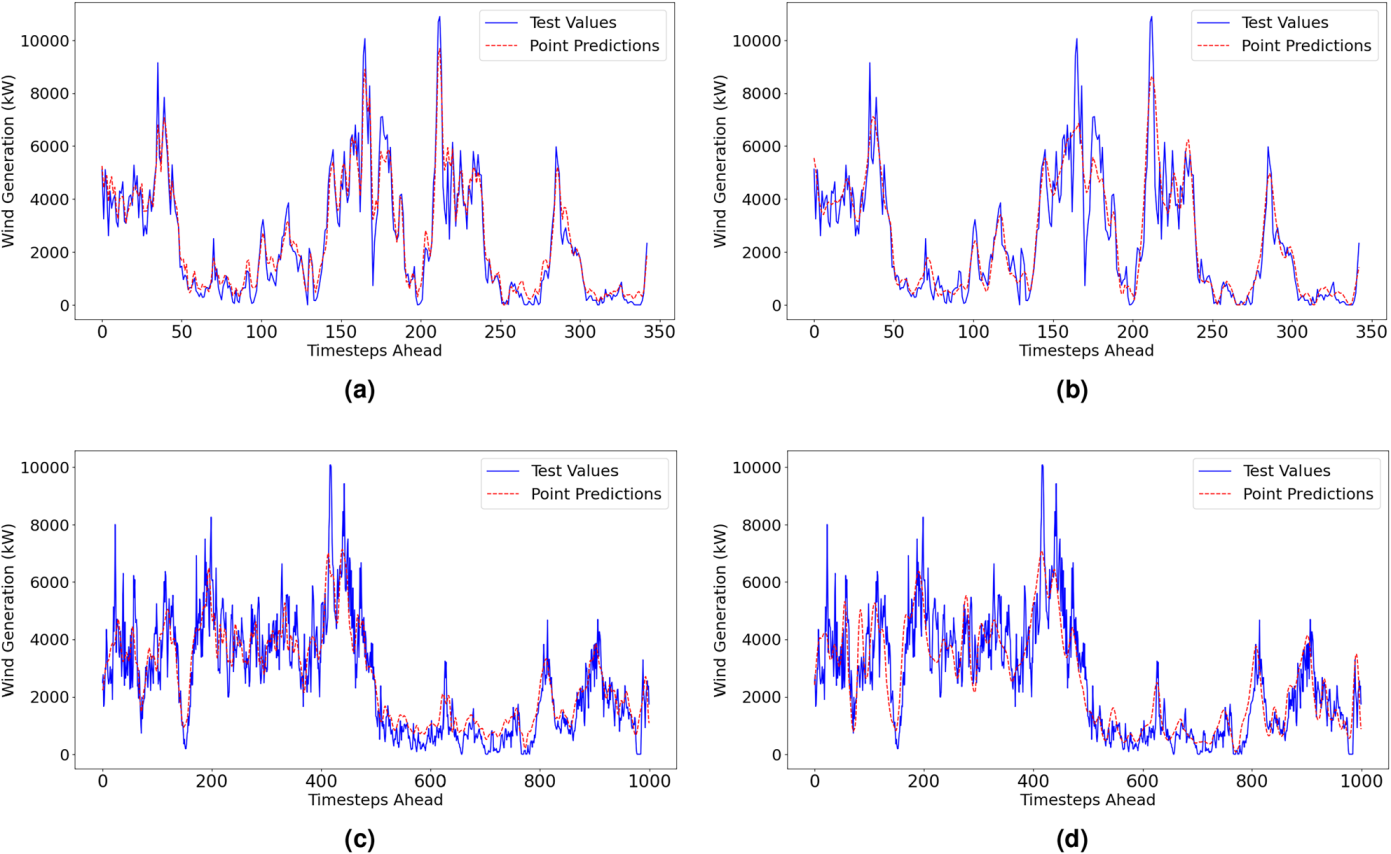
Deterministic results for the Kelmarsh dataset.

Deterministic forecasting performance metrics for the Kelmarsh wind farm dataset are shown in Table 6. Visualisations of the point estimate performance are shown in Fig. 9. The low values for *NMAE*, *NRMSE*, *MAPE* and the values close to 1 for *IA* indicate that the point forecasts have a high level of accuracy, particularly for the low-resolution data. As with the Penmanshiel dataset, the VMD-FFNN with low-resolution data was the most accurate forecasting method.

The *NBias* scores for the EEMD-FFNN method are closer to zero than the *NBias* scores produced by the VMD-FFNN method. The opposite is true for the *NSDE*, where scores are higher for the EEMD-FFNN method than the VMD-FFNN scores. This indicates that the EEMD-FFNN method has a more significant random error component, and the VMD-FFNN method has a more significant systematic error component. A similar trend is reflected in the deterministic results from the Penmanshiel dataset (Table 5).

**Table 6:** Point Estimate Results—Kelmarsh Wind Farm.

Forecast horizon = 6h ahead						
Method	NMAE(%)	NRMSE(%)	MAPE(%)	NBias(%)	NSDE(%)	IA
Low resolution dataset (1-hr resolution)						
VMD–FFNN	3.423	4.662	21.56	-1.10	4.53	0.9819
EEMD–FFNN	4.540	6.666	26.90	-0.65	6.63	0.9631
High resolution dataset (10-min resolution)						
Method	NMAE(%)	NRMSE(%)	MAPE(%)	NBias(%)	NSDE(%)	IA
VMD–FFNN	5.388	7.184	26.59	-0.89	7.13	0.9532
EEMD–FFNN	5.996	8.571	28.62	-0.20	8.57	0.9366

### Probabilistic results

#### Penmanshiel wind farm: prediction intervals

**Fig. 10:**
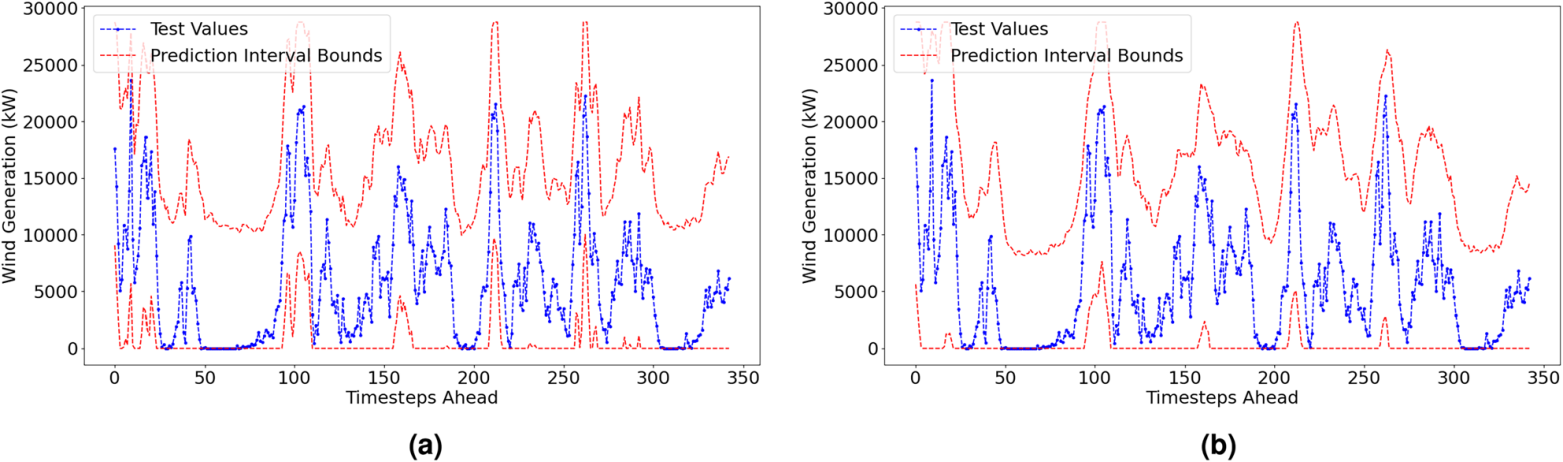
Probabilistic results for the Penmanshiel dataset.

The probabilistic performance metrics for the Penmanshiel wind farm dataset are shown in Table 7. Visualisations of the prediction interval forecasts are shown in Fig. 10 using the low resolution data (see 3.2 Experimental design: forecasts & errors).

Both methods achieved full reliability (values of 100% for *PICP*). The values for *PINAW* and *CWC* are similar between the Irish and Penmanshiel datasets; the prediction intervals are deemed to be sufficiently narrow to be considered informative probabilistic metrics. The *IS* is an order of magnitude larger for the Penmanshiel dataset than in the Irish dataset for both forecasting methods. This is purely due to the difference in capacity of the Penmanshiel wind farm and the Irish wind farm as the *IS* metric is not normalised by capacity.

**Table 7:** Prediction interval results—penmanshiel wind farm.

Confidence level: 95%					
Forecast horizon = 6h ahead					
Low resolution dataset (1-hr resolution)					
Method	PICP (%)	PINAW (%)	CWC	ACE	IS
VMD–FFNN	100.00	54.83	0.548	0.050	-1576.42
EEMD–FFNN	100.00	55.64	0.556	0.050	-1599.57

#### Kelmarsh wind farm: prediction intervals

**Fig. 11:**
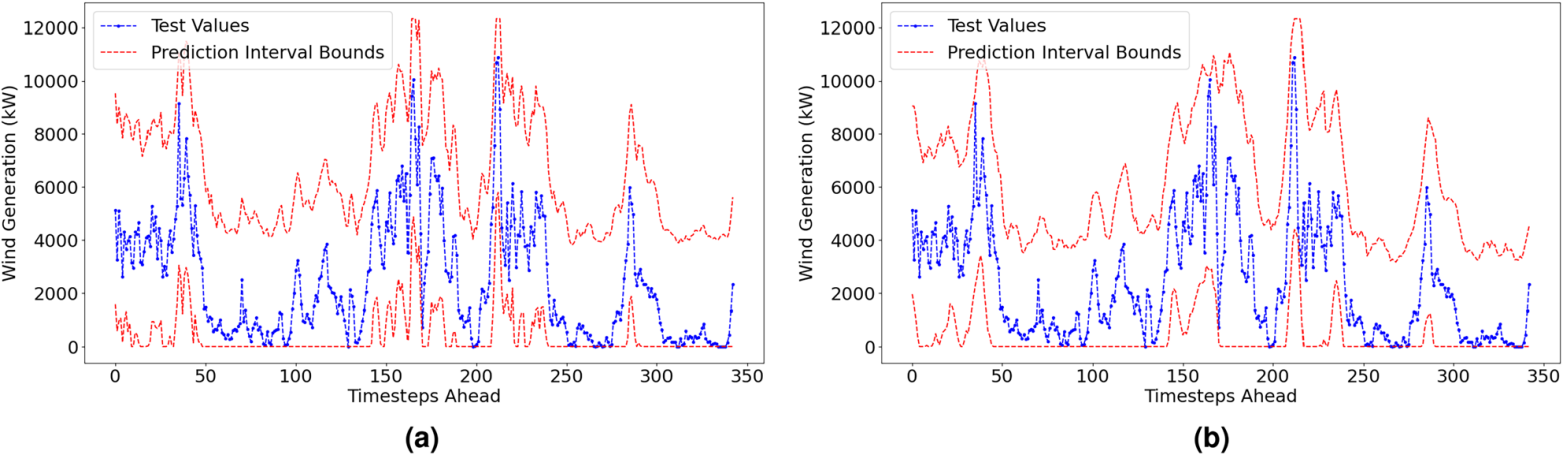
Probabilistic results for the Kelmarsh dataset.

The probabilistic performance metrics for the Kelmarsh wind farm dataset are shown in Table 8. Visualisations of the prediction interval forecasts are shown in Fig. 11. Again, the low resolution data *only* is used. As for the Penmanshiel dataset, both methods achieved full reliability (*PICP* values at 100%). The prediction intervals are the narrowest observed across the three datasets; the *PINAW* values are at least 5% lower than those produced for the Penmanshiel and Irish dataset. The EEMD-FFNN method produced a slightly narrower prediction interval than the VMD-FFNN method; there is a difference of $\sim$100 in the *IS* score between the two methods.

**Table 8:** Prediction interval results—kelmarsh wind farm.

Confidence level: 95%					
Forecast horizon = 6h ahead					
Low resolution dataset (1-hr resolution)					
Method	PICP (%)	PINAW (%)	CWC	ACE	IS
VMD–FFNN	100.00	49.72	0.497	0.050	-613.10
EEMD–FFNN	100.00	47.42	0.474	0.050	-584.64

### Portability of results

The influence of model hyperparameters on the forecasting accuracy is investigated to understand portability of results from one farm to another by computing how performance metrics respond to changes in a given hyperparameter, and how sensitive they are to these changes. The Penmanshiel and Kelmarsh wind farm datasets are used here. Four different hyperparameters are investigated,

1. Number of modes
2. Number of steps used at input in the FFNN
3. Number of training datasets used
4. Number of training simulations

**Fig. 12:**
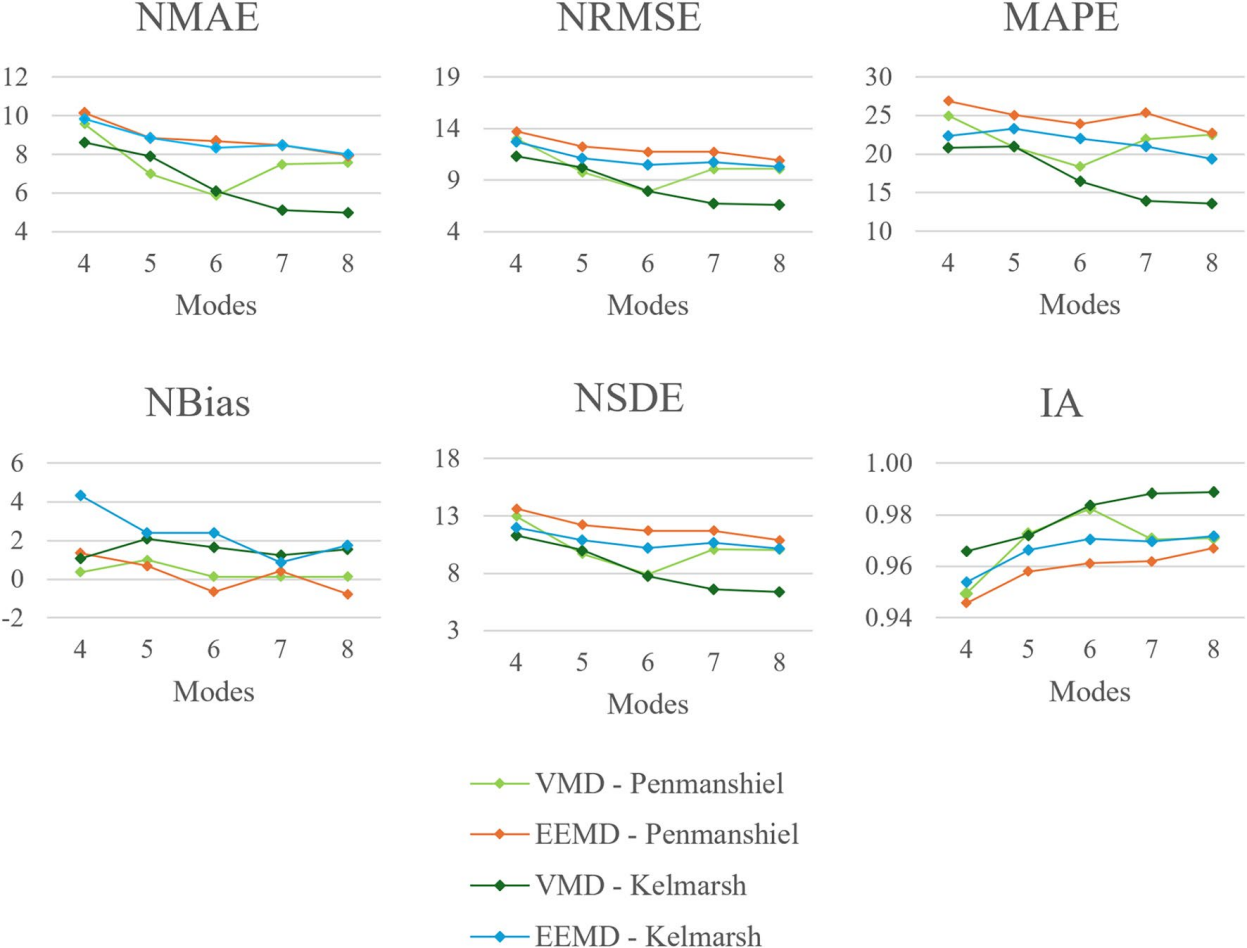
Changes in deterministic performance metrics with number of modes.

**Fig. 13:**
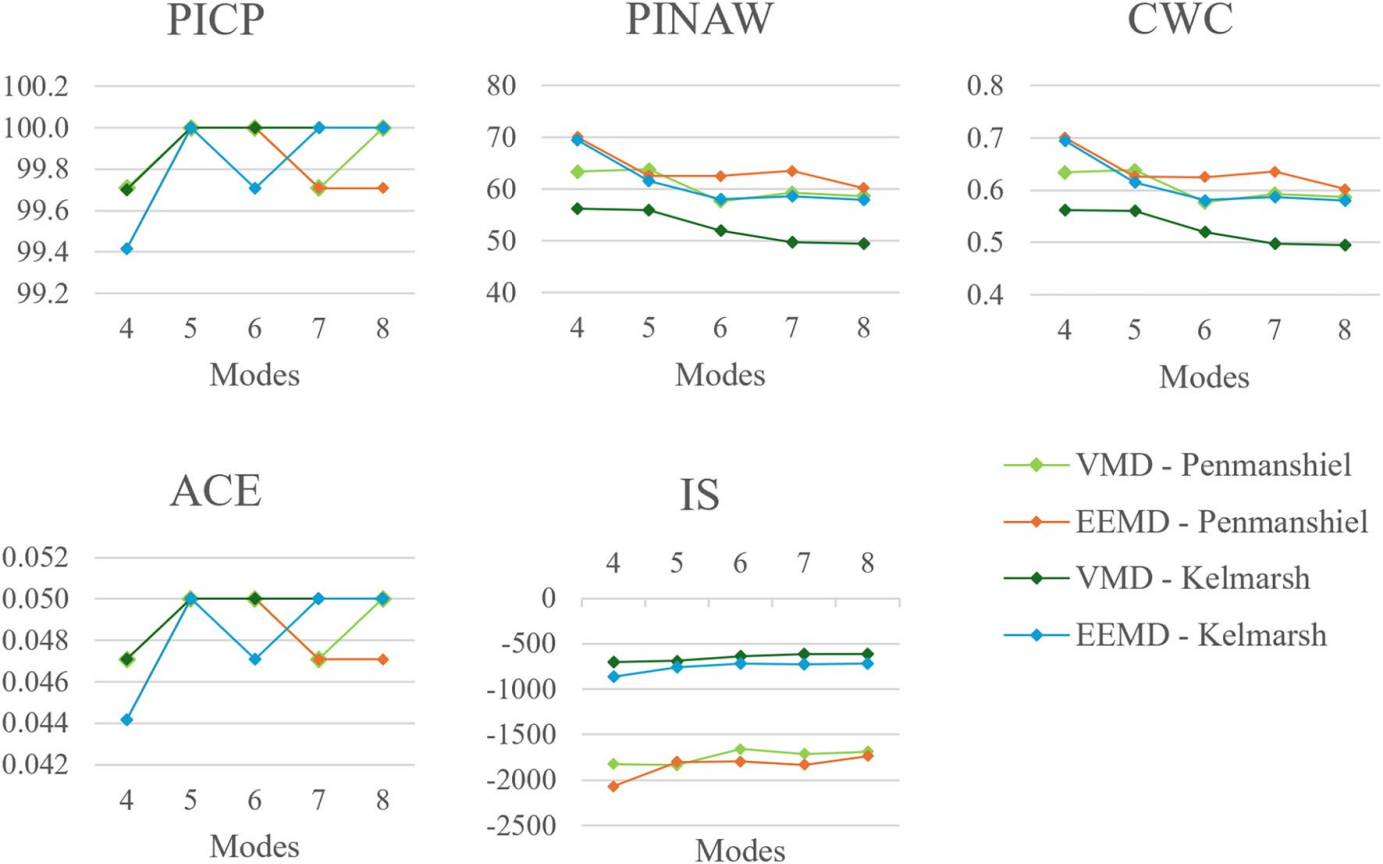
Changes in probabilistic performance metrics with number of modes.

**Fig. 14:**
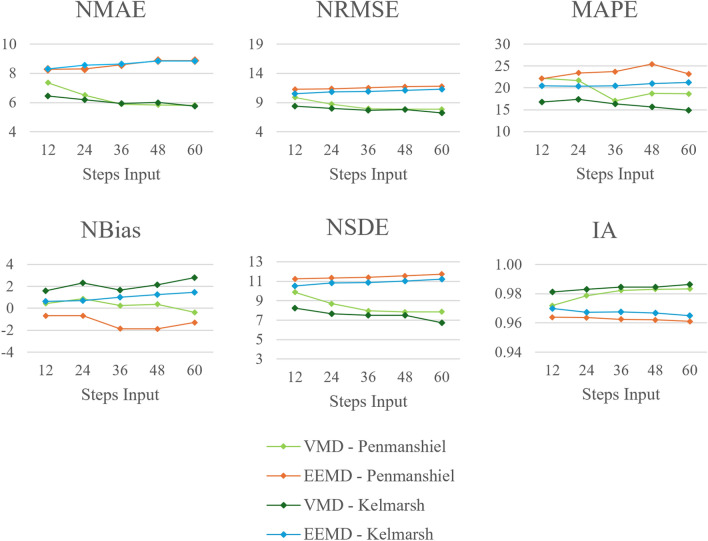
Changes in deterministic performance metrics with number of steps used at input.

**Fig. 15:**
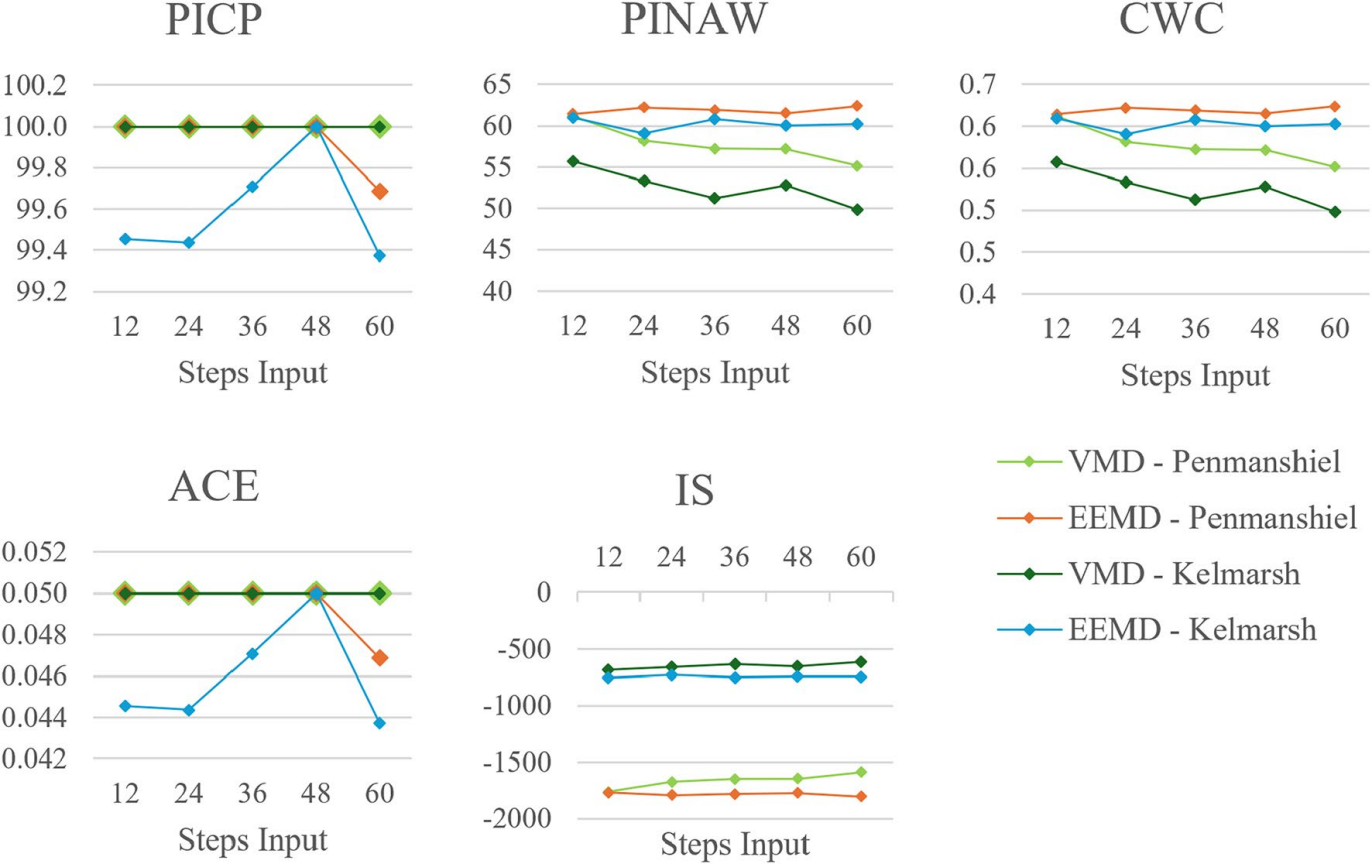
Changes in probabilistic performance metrics with number of steps used at input.

**Fig. 16:**
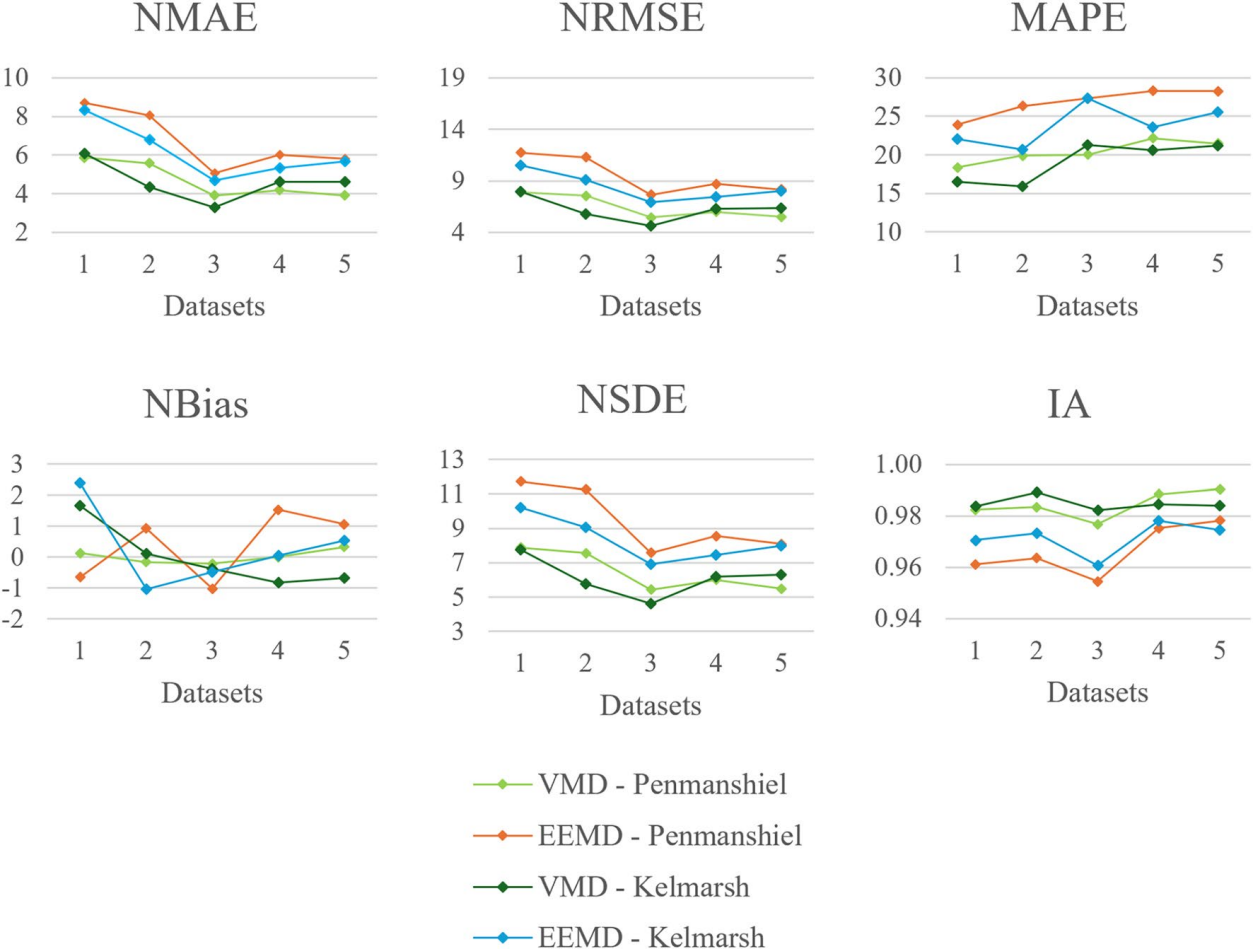
Changes in deterministic performance metrics with number of training datasets.

**Fig. 17:**
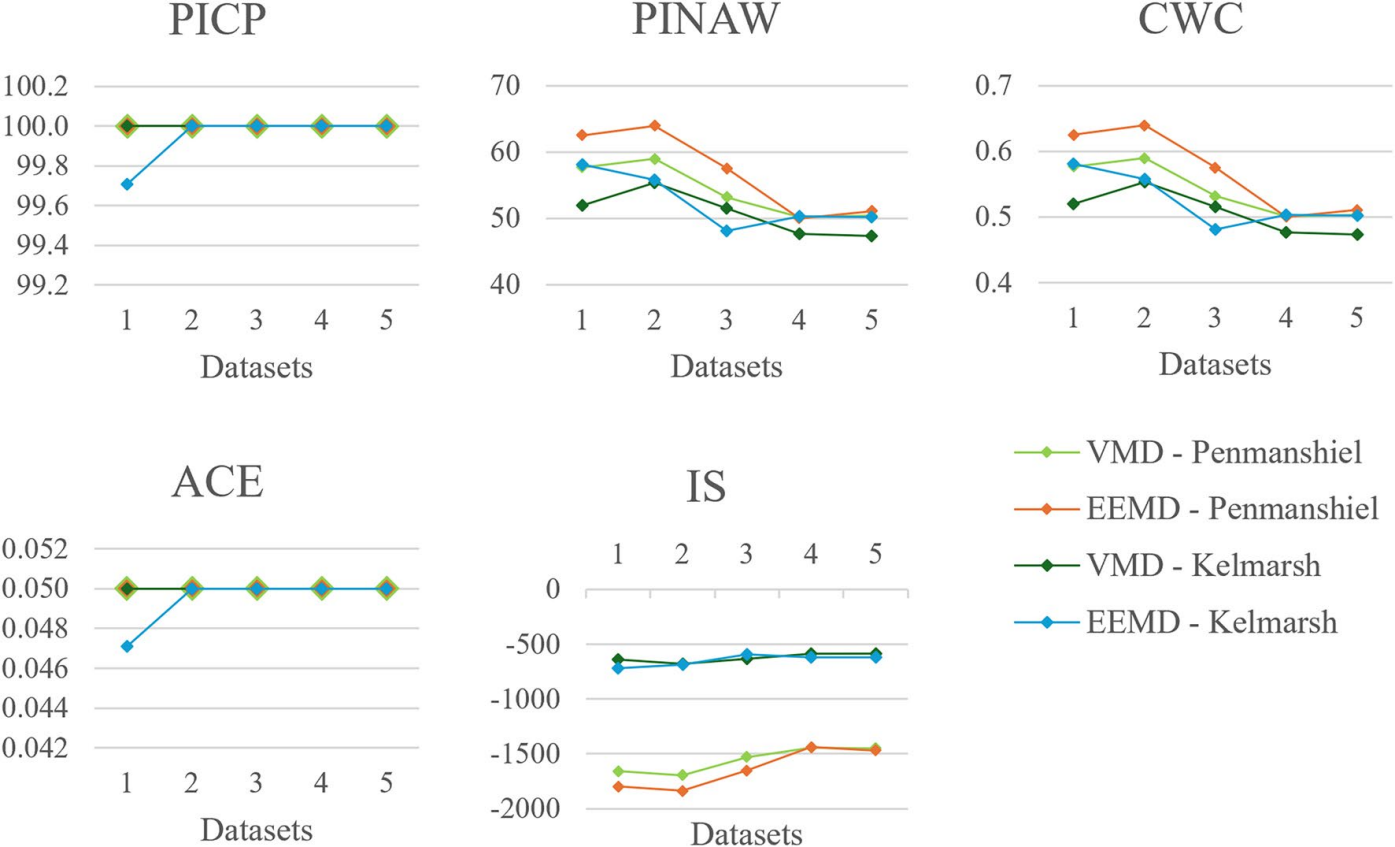
Changes in probabilistic performance metrics with number of training datasets.

**Fig. 18:**
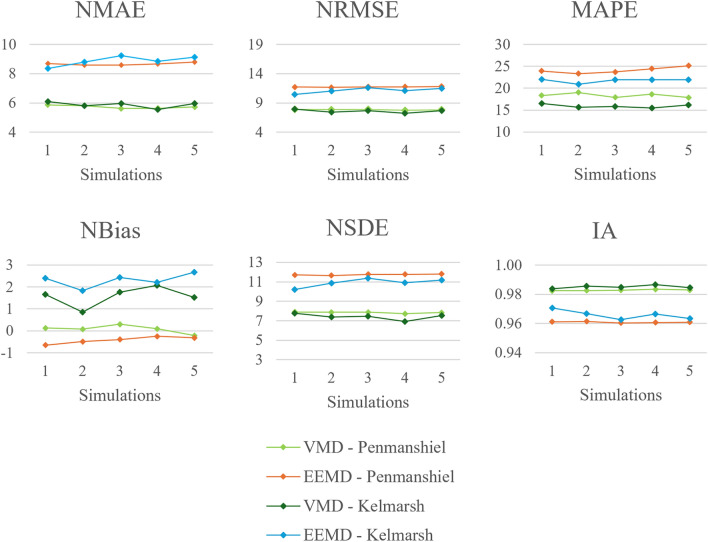
Changes in deterministic performance metrics with number of training simulations.

**Fig. 19:**
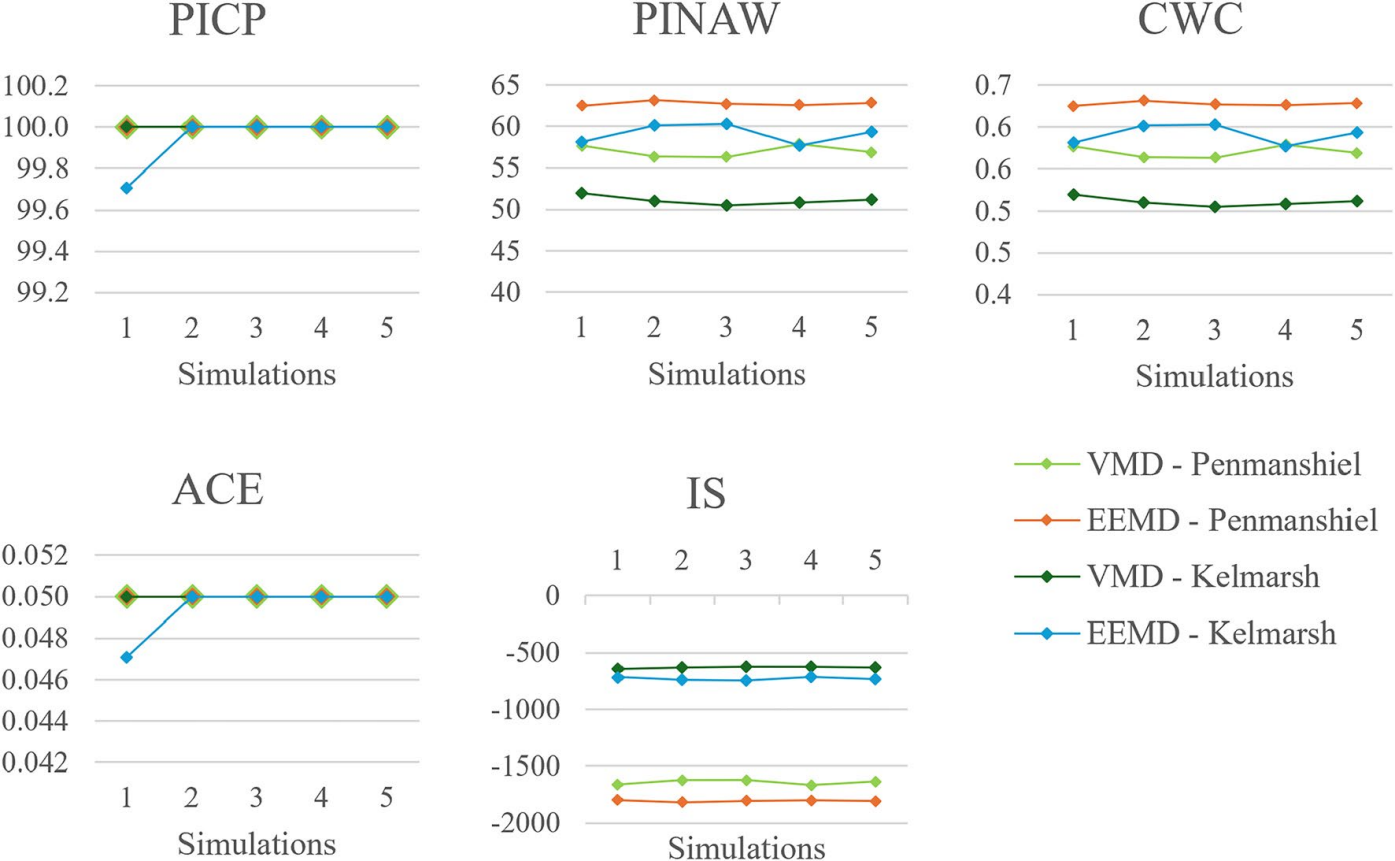
Changes in probabilistic performance metrics with number of training simulations.

Where one hyperparameter is altered, all other hyperparameters are kept constant. The base value for each hyperparameter in the models are described in Table 9. The base values (usually the mean of the range of values examined) used are typical for machine learning applications and were chosen to make the portability assessment computationally efficient, produce accurate forecasts at a reasonable time horizon, and to avoid skewing of the portability assessment results. The vertical axis has been scaled down several values in Figs. 12, 13, 14, 15, 16, 17, 18 and 19, so that it starts at a non-zero value to enhance clarity and readability. So, the actual differences in performance are often not as pronounced as they visually appear in these figures.

**Table 9:** Base hyperparameter values.

Hyperparameter	Base value
Data resolution	1-hour
Number of decomposition modes	6
Number of steps input at FFNN	36
Number of datasets	1
Number of simulations	1
Number of steps output at FFNN	6
Number of neurons in FFNN	10
Training epochs	100
Training batch size	64
Forecasting horizon (timesteps)	1, 2, 3, 4, 5, 6

#### Number of modes

Performance metrics, varying with the number of modes used in signal decomposition, are presented in Figs. 12 and 13. Errors in point predictions overall tend to reduce with a greater number of modes. This is evident in the *NMAE*, *NRMSE*, *NSDE*, and *MAPE*, where the values tend towards a minimum when a higher number of modes are used. The same trend is seen for the *IA*, where values tend towards 1 for a higher number of modes. There is one exception to this trend, where the VMD-FFNN method with the Penmanshiel dataset has the most accurate performance when six modes are used.

While the accuracy tends to improve significantly when the number of modes is increased from four to six, the improvement in accuracy slows when increasing from seven to eight modes. Overall, the forecasting performance appears to be sensitive to a low number of modes and less sensitive to a higher number, indicating that it is adequate for a limited number of modes to adequately describe the underlying processes linked to wind power generation for the site.

The values for *PICP* and *ACE* tended to be close to or at full reliability. However, these values are slightly lower when four modes are used. The values for *PINAW*, *CWC*, and *IS* reduce with a larger number of modes, although the reduction appears to plateau somewhat at a higher number of modes. The prediction intervals become more reliable and narrower with an increase in the number of modes, though this enhancement reaches a saturation point at a higher number of modes.

#### Number of steps input

Performance metrics, varying with the number of steps used at input in the FFNN, are presented in Figs. 14 and 15. The VMD-FFNN and EEMD-FFNN methods exhibit opposite trends when increasing the number of steps input. The accuracy of the VMD-FFNN method improved, while the accuracy of the EEMD-FFNN method reduced, although the changes are relatively minor and the accuracy is quite stable across variations in the number of steps input for both methods. The *NBias* also remains reasonably steady across variations in the number of steps input, indicating that there is minimal variation in the systematic component of the error.

Three of the four methods maintained almost 100% reliability across variations in number of steps input however, the EEMD-FFNN method with the Kelmarsh dataset is slightly less reliable given the lower values for *PICP* and *ACE*. For the EEMD-FFNN method, the values for *PINAW* and *CWC* are quite stable, fluctuating around a value of $\sim$61% and $\sim$0.61, respectively. For the VMD-FFNN method, the values for *PINAW* and *CWC* tended to reduce with a greater number of steps input. There is a significant difference in the values for *IS* for the two datasets, this is due to the difference in total capacity of the two datasets.

#### Number of training datasets

Performance metrics, varying with the number of datasets used for training, are presented in Figs. 16 and 17. The five datasets used are each a year long, they are sampled from the total dataset which is two and a half years in length (see Table 1).

The *NMAE*, *NRMSE* and *NSDE* tend to reduce between one and three datasets, values rise slightly for four and five datasets. A similar trend is reflected in the values for *IA*. The *MAPE* increases with a greater number of datasets. This is somewhat unexpected given that other metrics, such as the *NMAE*, suggest that the accuracy continues to improve with more training datasets. This discrepancy can be attributed to the addition of a larger quantity of data; training models with a variety of data can sometimes lead to an apparent reduction in accuracy, yet the models ability to generalize to new data is improved.

The values for *PICP* and *ACE* indicate full reliability for all method configurations except the EEMD-FFNN method with the Kelmarsh dataset, which was marginally lower. The *PINAW*, *CWC* and *IS* indicate that the sharpness of the prediction interval improves significantly when a greater number of training datasets are used. The sharpness of the prediction intervals are excellent when four and five training datasets are used.

Using a greater number of datasets adds additional training execution time. However, this added computational load is in direct exchange for improved forecasting performance. Additionally, using more datasets in model training gives models a greater variety of data, which is key to improving the ability of a model to adapt to new data.

#### Number of training simulations

Performance metrics, varying the number of training simulations, are presented in Figs. 18 and 19. Both deterministic and probabilistic metrics show minimal change when varying the number of simulations. The forecasting accuracy appears *not* be sensitive to the number of training simulations employed. This insensitivity to hyperparameter configuration is also seen for the number of steps input. In comparison, the forecasting accuracy is more sensitive to changes in the number of modes and number of datasets used.

Running more training simulations using the same dataset thus has minimal effect on modelling accuracy. Additional training simulation execution time can therefore be avoided without compromising forecasting accuracy. Furthermore, these results reiterate the importance of using a variety of high quality data from different sources to achieve accurate and robust forecasts.

In general, there is a trade off between training execution time (and therefore, computational load) and improving the accuracy of forecasts when changing hyperparameter configurations. This applies in particular to hyperparameters where the performance is sensitive to changes. This should be considered as part of model calibration. In future, these portability calibrations and results can be scaled to large projects by considering a large number of wind farms along with model types, hyperparameters, input steps, neuron numbers, training epochs and batch size.

## Discussion

The portability of model hyperparameters for deterministic and probabilistic wind power forecasting at farm level is considered by comparing the VMD-FFNN and EEMD-FFNN hybrid methods, for three different wind farms at a 6-hour ahead forecasting horizon.

Several findings emerged from the experimental results. Firstly, it is found that forecasting performance is not significantly dependent on the number of training simulations or number of input steps applied in the model. This is a useful result, as it confirms that these aspects of the model configuration are less significant when defining calibrations for larger wind farm fleets or regions. Additionally, a low number of modes in the decomposition step is adequate for good prediction, beyond a threshold of ~4 modes. That said, the number of modes used requires calibration for deterministic or probabilistic estimates depending on the wind farm. Training in diverse and actual datasets improved the resilience of the models. This is a valuable insight as this enables models to better capture and respond to the unique characteristics of new wind farms.

These findings can be utilised for more tailored model calibration for other wind farm sites. It demonstrates that it is possible to define forecasting models for a larger set of farms based on the calibration of one farm, which share similar attributes as evidenced by performance sensitivity of hyperparameters. Site-specific calibrations can lead to grouping of regions or dataset cohorts attributed to a forecasting type. For example, the calibration of a new Irish wind farm may be via a ’Kelmarsh-type’ configuration, bypassing significant computing or calibration efforts. This can enable the scaling of forecasting accuracy as described in the introduction to this paper.

With enhanced calibration methods, greater prediction accuracy at a wind farm fleet level is enabled, providing significant benefits. This includes enhanced operation of electricity transmission systems by improving unit commitment and economic dispatching processes and aiding the identification of network congestion and voltage violation. Additionally, this can improve strategic bidding and trading regimes used in electricity markets, bolster financial performance, and help to avoid market participation costs such as those associated with balancing actions.

The experimental results offer opportunity for further study and the development of practical applications. With greater availability of open-source wind power data, a portability framework can enable decisions at a regional level and create guidelines for site-specific calibrations, with comparability of different sites. Currently, the confidential and proprietary nature of wind power datasets is a barrier to a more unified and cohesive forecasting approach, such as the proposed portability framework. A unified forecasting framework and wind power dataset repository can lead to a resilient and stable way of managing large wind power systems and their demands, including enhancement of lifetime operations and accurate power forecasting.

The significance of the portability findings are enhanced with further complementary study. By examining other datasets, models, and model hyperparameters (such as training epochs, training batch sizes, etc) under the same portability framework, the potential to define calibrations for larger wind farm sets can be realised.

## Data Availability

The Penmanshiel and Kelmarsh wind farm datasets analysed are publicly available in the Zenodo repository, or via the following links, https://zenodo.org/records/8252025, https://zenodo.org/records/8253010. The Irish dataset analysed is not publicly available due to confidentiality reasons but is available on reasonable request by contacting Vikram Pakrashi on Vikram.Pakrashi@ucd.ie.
